# Composite vector formulation for multiple siRNA delivery as a host targeting antiviral in a cell culture model of hepatitis C virus (HCV) infection†

**DOI:** 10.1039/c6tb01718e

**Published:** 2017-01-09

**Authors:** E. Crouchet, R. Saad, C. Affolter-Zbaraszczuk, J. Ogier, T. F. Baumert, C. Schuster, F. Meyer

**Affiliations:** a Inserm, U1110, Institut de Recherche sur les Maladies Virales et Hépatiques, 67000 Strasbourg, France; b Université de Strasbourg, 67000 Strasbourg, France; c Inserm, U1121, Biomatériaux et Bioingénierie, FMTS, 67000 Strasbourg, France; d Institut Hospitalo-Universitaire, Pôle Hépato-digestif Hopitaux Universitaires de Strasbourg, 67000 Strasbourg, France; e Institut Hospitalo-Universitaire, Pôle de Médecine et Chirurgie Bucco-dentaires, Hôpitaux Universitaires de Strasbourg, 67000 Strasbourg, France

## Abstract

Hepatitis C virus (HCV) infection is a major cause of chronic liver disease and cancer worldwide. RNA interference (RNAi)-based gene therapies have emerged recently as a promising tool to treat chronic viral infections. Indeed, small interfering RNAs (siRNAs) provide an opportunity to target host factors required for the viral life cycle. In this study, we evaluated a novel nanovector-based approach for siRNA delivery in a model of chronically infected hepatic cells. We designed original composite nanoparticles by coating the calcium phosphate core with siRNAs targeting HCV host-factors and pyridylthiourea-grafted polyethyleneimine (πPEI). Using combinations of different siRNAs, we observed an efficient and prolonged decrease of HCV replication. Moreover, we showed that the layer-by-layer technique of coating applied to our nanoparticles triggers a sequential release of siRNAs acting on different steps of the HCV life cycle. Together, our results demonstrate the efficacy of these nanoparticles for siRNA delivery and open new perspectives for antiviral therapies.

## Introduction

Chronic hepatitis C virus (HCV) infection is a major cause of chronic hepatitis, liver cirrhosis and hepatocellular carcinoma worldwide.^1^ While recently clinically licensed direct-acting antivirals are expected to cure the large majority of infected patients, some challenges remain for difficult-to-treat patient subgroups.^2^ RNA interference (RNAi)-based gene therapies have recently emerged as a promising tool to treat chronic viral infections.^3–5^ However, considering the high mutation rates and the number of genotypes and sub-genotypes of RNA viruses such as HCV, targeting viral sequences remains challenging. The recent development of host targeting agents (HTAs), targeting host factors required for viral propagation, constitutes an interesting alternative to overcome viral mutation and resistance to treatment.^6^ Delivery of HTAs using the nanoparticle technology offers the opportunity to provide an effective and innovative strategy to widen the arsenal of antiviral therapy. From a biomaterial point of view, RNAi-based gene therapy allows to diversify siRNA sequences without modifying the overall chemistry of the molecule, resulting in a highly versatile molecule targeting different host factors with the same delivery strategy.^7,8^ Previous studies on the use of siRNAs have put emphasis on several advantages when working with such molecules. Firstly, using cocktails of different siRNAs targeting the same gene allows increasing the global siRNA concentration without reaching the threshold of off-target effects.^9,10^ Secondly, regarding antiviral therapy, it helps to treat multiple viral strains and sub-types. Several studies using both *in vitro* and *in vivo* models of HCV infection, showed that repeated treatments with two siRNAs were better than a single siRNA treatment at minimizing the development of escape mutants, resulting in a rapid inhibition of viral replication.^11,12^ Moreover, targeting various HTAs would be advantageous by controlling different steps of viral infection, from viral entry to cell re-infection.^6,13^ Finally, previous studies have demonstrated that, in contrast to the siRNA technology, the overexpression of exogenously introduced shRNA competes with that of endogenous miRNA and thus leads to the saturation of the endogenous miRNA pathway, resulting in serious toxicity in the mouse liver and, in some instances, death.^14^ Taking into account all these data is helpful to determine specifications for future nanovectors that could be used for applications requiring delivery of multiple siRNAs in a controlled manner.

Several attempts have been made using different approaches of vector formulations. Studies have been performed on siRNA modification as well as on nanovector formulation. In 2014, Sajeesh *et al*. showed that tripodal RNA structures complexed with galactose-modified polyethylene imine (PEI) could generate effective RNAi-mediated gene silencing in experimental mice models.^15^ Moreover, Lee et *al*. demonstrated that coating nanoparticles by alternative adsorption of polymers and siRNAs is a successful strategy to obtain a sustained delivery of siRNAs *in vivo* with a prolonged action.^16^ We previously developed a composite nanoparticle model based on the same approach. We chose to use calcium phosphate nanoparticles because they have good biodegradability and biocompatibility *in vivo*. Moreover, these nanoparticles are more stable than other vesicular vectors such as liposomes.^17^ Calcium phosphate nanoparticles were, previously, successfully coated with siRNAs and a tyrosine-modified PEI (PEI-Tyr) by alternative adsorption. These particles showed a high gene silencing efficacy *in vitro* and *in vivo* in a murine model of xenograft tumour.^18^ In the present study, we tested similar composite nanoparticles coated with siRNAs and pyridylthiourea-grafted polyethylenimine (πPEI) in a chronic HCV infection model. This polymer has a lower toxicity compared to PEI-Tyr and allows a similar siRNA complexation.^19,20^ Using nanoparticles coated with different siRNAs targeting host factors involved in the viral life cycle, we observed a great decrease of HCV replication over 10 days. Moreover, we demonstrated that the layer-by-layer coating technique proposed here causes a delayed delivery of the siRNAs. Altogether, these results pave the way to a more comprehensive use of such nanodevices in antiviral therapy.

## Results and discussion

### Particle formulation and characterization

The particle formulation (CPNp(πPEI/siRNA)_2.5_) was performed as previously described, using an alternate deposition of first πPEI and then siRNAs directly after calcium phosphate particle (CPNp) precipitation.^18^ 1.3 μg of siRNA per layer has been used for the coating of the nanoparticles. The concentration of πPEI added for the coating of the particles represents a N/P ratio = 3 (N corresponding to moles of the amine groups of polymers and P to the moles of phosphate groups of siRNAs).^18^ The average hydrodynamic diameter measured by dynamic light scattering (DLS) after coating is 83 ± 1 nm with a positive charge of 31 ± 5 mV. The particle size was confirmed by transmission electron microscopy (TEM) imaging (Fig. S1, ESI†). Moreover, we observed that the morphology of the core particles is the same before and after coating (Fig. S1, ESI†). In contrast, particles generated by alternate deposition of first siRNAs and then πPEI showed an increase of hydrodynamic diameter up to 100 nm, reflecting severe aggregation (data not shown). This observation points out the importance of alternate deposition sequence. With our previous formulation, we obtained particles with a hydrodynamic diameter of 56 nm using PEI-Tyr as the coating agent.^18^ This difference could be explained by a different organisation of the πPEI polymer on the calcium phosphate core. Moreover, with our new formulation (CPNp(πPEI/ siRNA)_2.5_), we have more PEI present on the particle surface compared with our previous formulation (CPNp(siRNA/PEIY)_2_).^18^

We then analysed the stability of the coated particles for 8 days by DLS measurements. We observed that the particle diameter remains stable over time in acetate buffer, indicating that coating with polymer multilayers stabilizes the particles (Fig. S2, ESI†). Moreover, no siRNA release was detected by electrophoretic mobility assay when the particles were incubated in water or in fetal bovine serum (Fig. S3, ESI†). Since no free siRNAs (displaying a different migration profile compared with complexed siRNAs) were detected, we assume that complete encapsulation of siRNAs was obtained. This result is in accordance with our previous data obtained using PEI-Tyr as the coating agent. Indeed, PEI-Tyr and πPEI were synthesized using the same 25 kDa branched PEI. Altogether, these results demonstrate that the particles remain stable over time.

πPEI toxicity was then determined using a 3-(4,5-dimethylthiazol-2-yl)-2,5-diphenyltetrazolium bromide (MTT) test in human hepatocellular carcinoma Huh7.5.1 cells. No toxicity was detected up to 375 μM πPEI. This concentration is way above the 100 μM πPEI classically used in our transfection experiments (Fig. S4, ESI†).

### *In vitro* particle internalisation

Particle internalisation is of paramount importance for the delivery and efficacy of siRNAs. The overall charge of πPEI causes non-specific interactions with the cytoplasmic membrane and promotes non-specific internalization. Moreover, the presence of a high tertiary amine density in PEI structure helps to trigger particle endocytosis by the so-called “proton-sponge” effect in which the buffering capacity of PEI induces endosomal disruption and prevents nucleic acids from lysosomal degradation. Indeed, during PEI endocytosis, ATPase proton pumps actively translocate protons from the cytosol into the endosomes, leading to endosome acidification. Because of its high buffering capacity, PEI becomes protonated and decreases endosome acidification. This phenomenon results in a continuous entry of protons in endosomes followed by a passive influx of chloride ions and water, leading to an osmotic swelling and endosome rupture.^21–23^ Despite the fact that the proton sponge effect remains the most accepted mechanism, it is intensively debated nowadays. Recently, Benjaminsen *et al*., demonstrated that PEI does not change the endolysosome pH after cellular internalization, making uncertain that the “proton sponge” effect is responsible for PEI escape. Nevertheless this result could be explained by an increased proton transport by V-ATPase that is able to overcome the PEI buffering capacity and that maintains acidic conditions in endolysosomes.^24^ Other authors argued that the “proton sponge” effect is not the leading mechanism for PEI endosomal escape, which could be deleterious to the cell. Another explanation could be that PEI escapes from lysosomes through membrane pores/holes triggered by an interaction between PEI and the membrane combined with membrane tension.^21,24^ The mechanism of PEI escape from the endosomal/lysosomal pathway is still elusive.

**Fig. 1:**
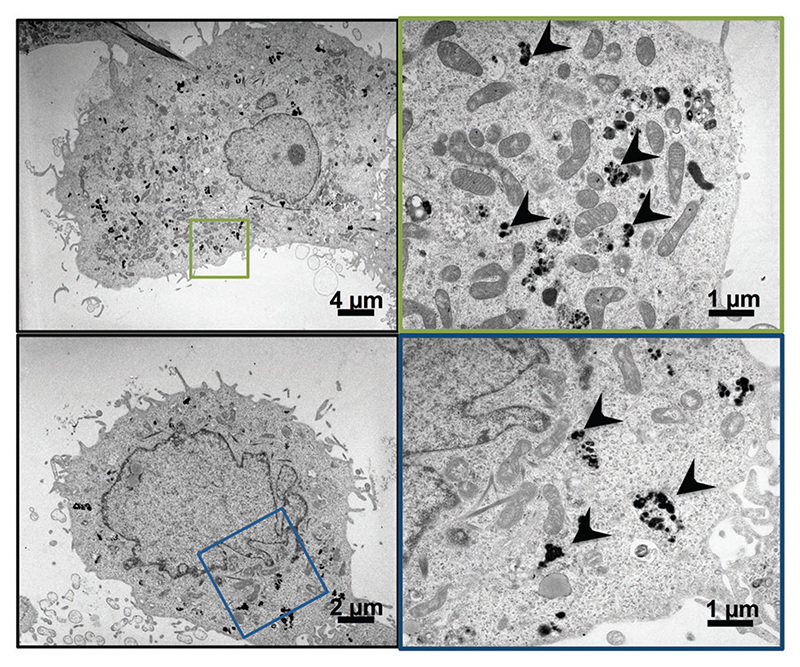
TEM (transmission electron microscopy) micrograph of Huh7.5.1 cells after 24 h (upper panels) or 48 h (lower panels) incubation with CPNp(πPEI/siRNA)_2.5_.

**Fig. 2:**
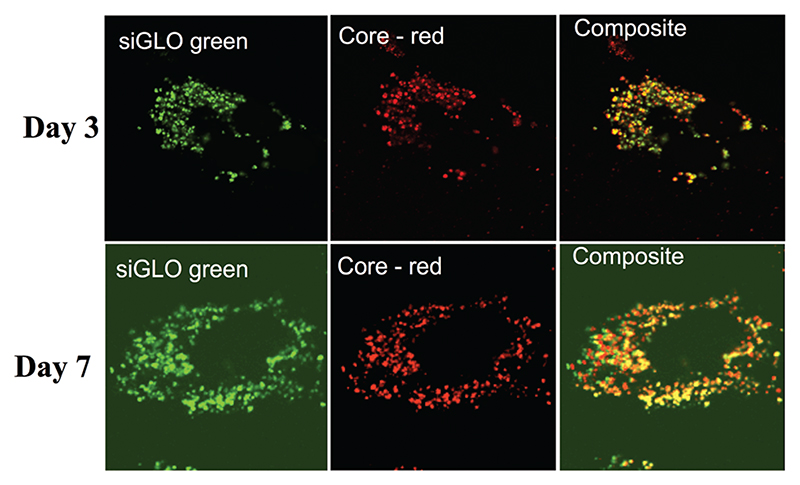
Confocal laser scan microscopy (CLSM) images of Huh7.5.1 cells after incubation with CPNp(πPEI/siRNA)_2.5_ showing the localisation of calcium phosphate core particles (red) and siRNAs (green). The images were taken at 63× magnification. Scale bars represent 5 μm.

Although there is no doubt that the particles are internalised, their intracellular localisation and fate are unclear. To address this question, we used TEM and confocal laser scan microscopy (CLSM) imaging. Huh7.5.1 cells were incubated with nanoparticles for 4 h. After thorough washing to discard all non-internalised particles, the cells were grown for an additional 24 or 48 h. TEM imaging of cells, 24 h and 48 h after particle endocytosis, shows that the particles are evenly distributed in the cytoplasm mainly as aggregates (Fig. 1). As expected, the particles are neither included in vesicles nor associated with membranes. However, the calcium phosphate core is the only part of the particles that can be visualised by TEM. In order to follow the presence of siRNAs, CLSM experiments were performed on Huh7.5.1 cells after incubation with nanoparticles containing alizarin complexone in the calcium phosphate core, and coated with FITC labelled siRNAs. Neither core particle nor siRNA labelling modifies the particle properties. As shown in Fig. 2, the core of the particles (red) as well as the siRNAs (green) are evenly distributed in the cell cytoplasm, partially co-localised, and can be visualised for at least 7 days. This demonstrates that the siRNAs coated on the particles are not released in the cells at once, since fluorescence-labelled siRNAs are observed to be co-localized with the core particles until 7 days post-treatment. However, it is not known if siRNAs complexed with πPEI are still available and/or released over a long period of time.

### SiRNA delivery and particle toxicity

**Fig. 3:**
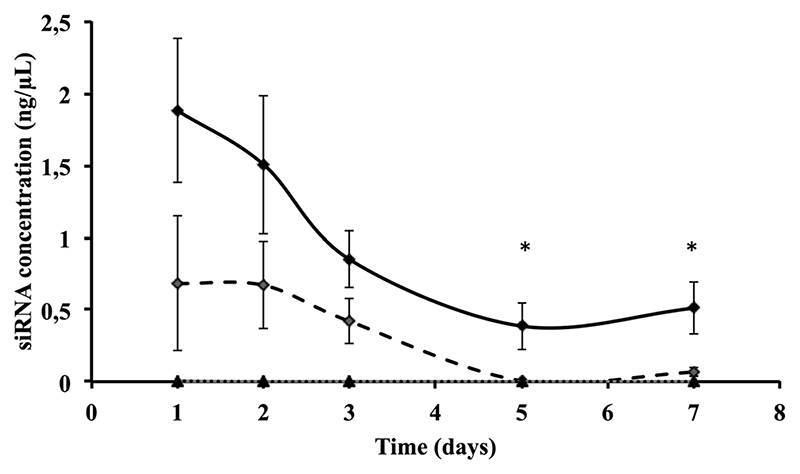
Intracellular siRNA quantification in Huh7.5.1 cells 24 h after incubation with 400 ng of CPNp(πPEI/siLuc)_2.5_ (black diamonds), 200 ng of CPNp(πPEI/siLuc)_2.5_ (open diamonds) and 400 ng of CPNp(πPEI/siGen1)_2.5_ (triangles), with siGen1 being a non targeting control siRNA. Quantification was performed by qRT-PCR using TaqMan^®^ small RNA assays (Life Technologies).

We then quantified intracellular siRNA delivery by composite nanoparticles in Huh7.5.1 cells, by quantitative RT-PCR. Our method allows only the detection of released siRNAs and not siRNAs complexed with πPEI. As shown in Fig. 3, a strong release of siRNAs was detected on the first two days after particle internalisation, followed by a decrease of the siRNA concentration detected. SiRNA delivery by our particles is related to a burst release following the particle entry. When cells are incubated with 200 ng of siRNAs no siRNAs were detected after 4 days. However, by increasing the siRNA concentration to 400 ng about 25% of siRNAs is still detectable after 4 days (Fig. 3). It is important to note that the remaining siRNAs detected might be related to the non-complete destruction of siRNA released in first instance. Our results suggest that increasing the particle concentration could lead to a prolonged siRNA delivery in cells and therefore a prolonged gene silencing. However, this result must be placed in parallel with the results obtained by CLSM. Indeed in Fig. 2, due to CLSM resolution, we can only detect FITC-labelled siRNAs that are still complexed with πPEI on the calcium phosphate nanoparticles. Moreover, particle aggregates are more easily detected compared with isolated particles. Increasing the particle concentration is so far limited by this aspect as particle or polymer complexes aggregated into the cells could display some toxicity.

**Fig. 4:**
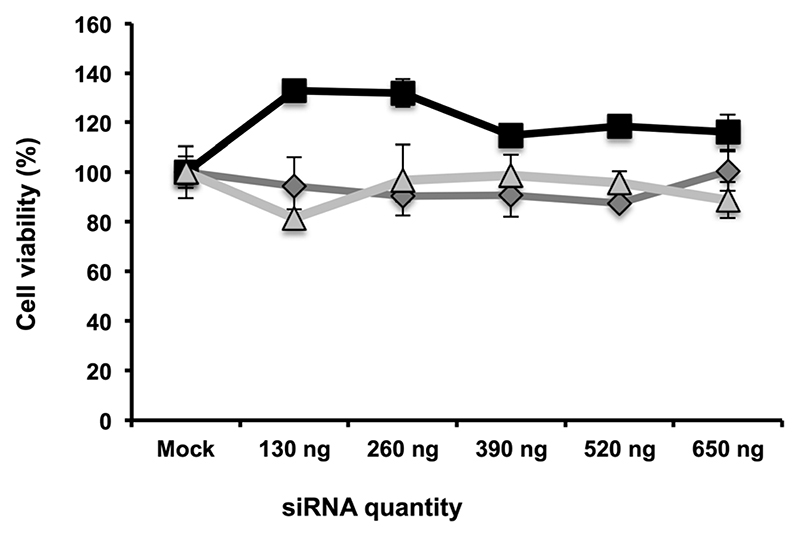
Quantification of Huh7.5.1 cell viability using a PrestoBlue test 24 h after incubation with CPNp(πPEI/siRNA)_2.5_ at various concentrations. The potential toxicity of different siRNAs used in this study was compared: siCD81 (grey diamond), siRACK1 (black square) and siApoE (white triangle).

Toxicity of such composite nanoparticles can be driven either by πPEI itself or by the siRNAs. Since πPEI was found to be innocuous at these concentrations (Fig. S4, ESI†), we tested in side-by-side different siRNAs hereinafter to evaluate whether the siRNA sequence itself could lead to cytotoxicity. To address this question, we performed a cell viability assay on Huh7.5.1 cells incubated with different concentrations of particles, coated with different siRNAs using the PrestoBlue^®^ test. The results presented in Fig. 4 show cell viability as a function of the siRNA quantity present on the particles for siRNAs targeting three different host factors. No toxicity was detected at any concentration tested, regardless of the siRNA sequence.

### Effect of composite nanoparticles on chronic HCV infection in human hepatoma cells

As a proof-of concept, we then assessed the efficacy of our particles in HCV-infected Huh7.5.1 cells.^25^ We used the Luc-Jc1 strain corresponding to a chimeric highly infectious HCV genome carrying the firefly luciferase reporter gene. This strain allows us to easily quantify viral replication, as luciferase expression is proportional to viral replication.^26^ Huh7.5.1 cells were electroporated with Luc-Jc1 RNA to obtain chronically HCV replicating cells. We designed nanoparticles containing 4 different siRNAs: one directly targeting viral RNA (siHCV) and three targeting host factors involved in different steps of the HCV life cycle (siCD81, siRACK1 and siApoE). siHCV targets the HCV internal ribosome entry site (IRES) sequence leading to a direct destruction of the viral RNA.^27^

siCD81 targets the tetraspanin CD81, a major actor of viral entry and therefore an important target to limit the reinfection process.^28^ SiRACK1 targets the receptor for activated C kinase 1 (RACK1) that has recently been proven to play a critical role in viral translation and replication, and therefore has been discovered as a target for broadly acting antivirals.^29^ Finally, siApoE targets apolipoprotein E (apoE), a key host factor involved in HCV entry, assembly and release of viral particles.^30–33^ First, we assessed nanoparticle efficacy in the HCV infectious model using particles coated with a single siRNA (Fig. S5, ESI†).

Cells transfection with nanoparticles leads to a decrease in HCV replication in a dose dependant manner at three days after transfection regardless of the target. siHCV and siRACK1 were the most efficient, with a decrease up to 95% (±1.2%) and 89% (±7.7%) of viral replication, respectively, at the highest siRNA concentration (650 ng). Under all the conditions tested, the highest concentrations were equivalent in efficacy to a transfection with a commercial transfection reagent (DF = Dharmafect) used as a positive control. More interestingly, the use of πPEI alone as a delivery platform turned out to be less effective as the nano-particles, since the presence of the calcium phosphate structure increases the inhibition of HCV replication. Our results are in accordance with a study published by Sokolova *et al*. showing that complexation of PEI with calcium phosphate particles improves siRNA delivery.^34^ As combining different siRNAs has been shown to increase antiviral efficacy,^11^ we prepared nanoparticles coated with two different siRNAs. HTAs constitute a promising strategy to treat HCV infection with low viral resistance to treatment.^6^ For these reasons, we chose to simultaneously target two host factors involved in different steps of the HCV life cycle by combining siRACK1 (viral translation/replication^29^) and siCD81 (viral entry and reinfection^28^). We observed that the siRACK1/siCD81 association exhibited an increased efficacy, especially at lower doses, compared with siRACK1 and siCD81 alone (Fig. S5, ESI†). For a final quantity of 130 ng of siRNA, the luciferase signal decreases up to 67% (±18.7%) for the siCD81/RACK1 mixture, instead of only 42% (±8.5%) with siRACK1 alone and 10% (±17.1%) with siCD81 alone.

Our previous experiments suggested that siRNAs are progressively released in cells up to 7 days. To understand if alternate layering changes the siRNA release sequence, we modified the particle coating by alternative deposition of both individual siRNAs, instead of using a mixture of siRNAs. We formulated 5 particles coated with different combinations of siRNAs, designed as follows:

CPNp(πPEI/siRACK1/πPEI/siCD81/πPEI),
CPNp(πPEI/siCD81/πPEI/siRACK1/πPEI),
CPNp(πPEI/siRACK1/πPEI/siCTRL/πPEI),
CPNp(πPEI/siCTRL/πPEI/siRACK1/πPEI),
CPNp(πPEI/siRACK1 + siCD81/πPEI/siRACK1 + siCD81/πPEI).

**Fig. 5:**
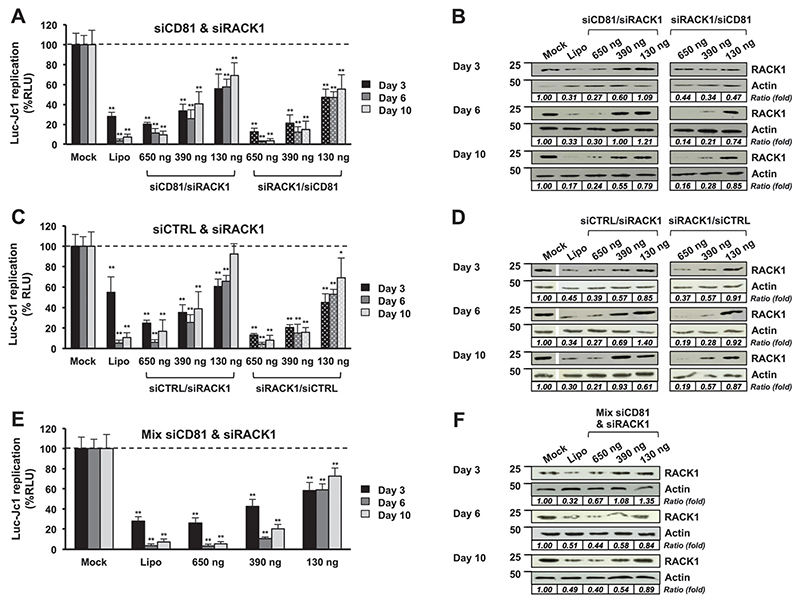
Effect of composite nanoparticles on HCV infection in Huh7.5.1 cells: HCV replicating cells were propagated in complete medium supplemented with 1% DMSO to slow down cell proliferation, and transfected with various types of particles coated by alternate deposition of siCD81, siRACK1 or a non-targeting siRNA (siCTRL). Different combinations of siRNAs were tested: siCD81/siRACK1 or siRACK1/siCD81 (A), siCTRL/siRACK1 or siRACK1/siCTRL (C) and a mix of siCD81 and siRACK1 (E). The particles were transfected to obtain 650, 390 or 130 ng of siRNA (final quantity). For each experiment, a control transfection was performed using the Lipofectamine RNAiMax (Lipo) transfection protocol, purchased from Life Technologies” (Carlsbad, CA, USA). Viral replication was assessed at 3, 6 and 10 days post-transfection by measuring the luciferase activity. The results are presented as percentage of luciferase activity relative to non-transfected HCV replicating cells (Mock = 100%). Means ± SD from three independent experiments performed in triplicate are shown. In parallel, silencing efficacy was assessed by the western blot analysis on the RACK1 protein (B, D and F). Relative quantifications (Image J software) are expressed as a ratio RACK1/actin (fold).

For example, in CPNp(πPEI/siRACK1/πPEI/siCD81/πPEI) particles, siRACK1 is close to the core particles (CPNps) and siCD81 is present at the particle surface. Thus, siCD81 could be released early after cell transfection, compared to siRACK1. In contrast, in CPNp(πPEI/siCD81/πPEI/siRACK1/πPEI) particles, siRACK1 is at the particle surface and siCD81 is close to the particle core. For a better understanding of our results, we abbreviated the different formulations by indicating first the siRNA present at the particle surface and then the second siRNA close to the particle core, *e.g*. “siCD81/siRACK1” and “siRACK1/siCD81” (Fig. 5).

All five types of particles were tested as described before. HCV replicating Huh7.5.1 cells were transfected with the particles at various concentrations. Two main parameters were recorded at 3, 6 and 10 days after transfection: luciferase activity as a marker of HCV replication (Fig. 5A, C and E) and RACK1 expression by western blot analysis (Fig. 5B, D and F). As observed previously, with a mixture of two siRNAs, a decrease of 80% (±2.1%) (D3), 89% (±4.6%) (D6) and 90% (±3.7%) (D10) of HCV replication is observed at the highest siRNA concentration (650 ng) (Fig. 5A). At the lowest siRNA concentration (130 ng), the sequential coated particles present a higher inhibition efficiency than the mixture-coated particles (compare Fig. 5A and E), meaning that the sequential coating presents a functional advantage.

A decrease of RACK1 protein expression was observed by the western blot for all types of particles. Interestingly, the silencing is maintained up to 10 days after transfection with the two highest concentrations. These results suggest a constant release of siRNAs over time. However, the sequence of the coating is important. By comparing RACK1 expression profiles and HCV inhibition profiles, we observed a difference in RACK1 silencing and HCV replication between siCD81/siRACK1 and siRACK1/ siCD81 particles. When siRACK1 is close to the core of the particle (siCD81/siRACK1), we observed a decrease of RACK1 expression three days after transfection at all concentrations, followed by a relapse from day 6, whereas when siRACK1 is at the surface of the particles (siRACK1/siCD81), we observed a significant decrease of RACK1 expression up to day 10. This is particularly obvious at an intermediate concentration of 390 ng (compare siCD81/siRACK1 and siRACK1/siCD81 in Fig. 5A and B). Such results indirectly prove that the coating process is important to adjust siRNA delivery, and were confirmed by another set of experiments using siRACK1 and siCTRL (compare siCTRL/siRACK1 and siRACK1/siCTRL in Fig. 5C and D). Moreover, we observed that siCD81/siRACK1 particles are less efficient to decrease HCV infection compared with siRACK1/siCD81 particles. All together, these results suggest that our nanoparticle formulation allows a sequential release of the siRNAs in cells and a sequential targeting of two different steps of the HCV life cycle. Moreover, we proved that targeting first HCV translation and replication with siRACK1 and then HCV reinfection with siCD81 in chronically infected cells constitutes a promising strategy to inhibit HCV propagation.

## Experimental

### Cell line

Human hepatoma Huh7.5.1 cells were propagated in Dulbecco’s modified Eagle’s medium supplemented with 10% of decomplemented fetal bovine serum, gentamycin (50 μg mL^–1^) and non-essential amino acids.

### RNA interference assays

Specific siRNA targeting CD81 (siCD81, L-017257-00), ApoE (siApoE, L-006470-00), the firefly luciferase (siLuc, D-001400-01) and non-targeting control siRNAs (siCTRL, D-001810-10-05; siGen1, D-001206-13) were purchased from Dharmacon Inc. (Chicago, Il, USA). The siRNA targeting HCV IRES sequence (siHCV331) was designed by Yokota *et al.^27^* (5’-GGUCUCGUAGACCGUGCACTT-3’). Specific siRNA targeting RACK1 (GNB2L1 silencer select, # 4392421) was purchased from Ambion, Life technologies™ (Carlsbad, CA, USA). Target gene expression was verified by the Western blot analysis as described previously.^32^

### Antibodies

Mouse monoclonal anti-β-actin (ab1906) was obtained from Abcam (Paris, France). The mouse monoclonal anti-core (MA1-080) antibody was obtained from ThermoFisher Scientific (Waltham, MA, USA). Mouse monoclonal anti-RACK1 (sc-17754) was obtained from Santa Cruz Biotechnology Inc. (Santa Cruz, CA 95060, USA). The secondary antibody anti-mouse IgG (NXA931) coupled with HRP was obtained from Amersham, GE Healthcare.

### HCV production and infectivity

The plasmid pFK-Luc-Jc1 (Luc-Jc1) construct has been previously described.^26^ Luc-Jc1 HCV RNA was obtained following T7 *in vitro* transcription of the plasmid pFK-Luc-Jc1. Luc-Jc1 is a chimeric HCV genome, which consists of J6CF structural protein segment and JFH1 (Japanese fulminant hepatitis 1) non-structural protein segment, and carrying the firefly-luciferase reporter gene.^25,26^

To obtain HCV replicating cells, Huh7.5.1 cells were electroporated with Luc-Jc1 viral RNA as described previously.^35^ Three days post-electroporation, Luc-Jc1 replication was assessed in cell lysates by measuring the luciferase activity.

### Preparation of calcium phosphate nanoparticles

CPNps were prepared by a wet chemical process using calcium acetate ((CH_3_COO)_2_Ca) (AR Aldrich) and sodium di-hydrogen phosphate (NaH_2_PO_4_) (AR, Aldrich). For the precipitation process, 50 mL of NaH_2_PO_4_ solution at 2 mM, heated at 60.2 °C, were added dropwise, at a rate of 2.5 mL min ^–1^, to 60 mL of (CH_3_COO)_2_Ca (2 mM) and the pH was adjusted to 5.15 by adding sodium acetate 5 mM/acetic acid, heated at 60 ± 2 °C under constant stirring. After precipitation the CPNp suspension was cooled down to 4 °C for conservation until use.

### siRNA and πPEI multilayer coating on nanoparticles

CPNps were coated by alternated deposition of siRNA and πPEI leading to a polyelectrolyte multilayer (πPEI/siRNA)_2,5_. The pyridylgrafted PEI (πPEI, 30% grafting) prepared, as hydrochloride salts, according to a described procedure from a 25 kDa branched PEI (40,872-7, batch09529KD-466, Sigma Aldrich St Quentin, France), was kindly provided by Dr Benoît Frisch (CNRS, University of Strasbourg, France).^18^ The CPNp suspension was kept at 4 ± 2 °C during the coating process. Before coating, the pH of the CPNp suspension was adjusted to 5.15. Under constant stirring, 11 μL of πPEI (10 mM N) per 1 mL of suspension was added. CPNps were incubated for 1 h in the presence of πPEI. Then, 1.3 μg of siRNA per mL of CPNp suspension (0.02% p/v) was added. CPNps were incubated for 1 h in the presence of siRNA. These steps were repeated 2 times to obtain a typical (πPEI/siRNA)2,5 coating at the surface of the nanoparticles. After completion of the coating, CPNp(πPEI/siRNA)_2,5_ were kept at 4 °C until use.

### DLS and zeta potential measurements

The apparent size and surface charges of the various coated nanoparticles were determined *via* dynamic light scattering (DLS) measurements using NanoZS apparatus (Malvern instruments, Paris, France) with the following specifications: sampling time 90 s; refractive index of the medium 1.3402; refractive index of particles 1.47; medium viscosity 1.145 cP and temperature 25 °C. Data were analyzed using the multimodal number distribution software included with the instrument. The measurements were performed on a nanoparticle solution corresponding to a siRNA quantity of 2.6 μg per mL of nanoparticles (1.3 μg of siRNA per layer).

### Electron microscopy

Huh7.5.1, seeded at 1 × 10^5^ cells per cm on glass coverslips (14mm diameter) 12 h prior to the experiment, were incubated with CPNp(πPEI/siRNA)_2,5_ at a concentration of 400 ng per well. At various times, cells were fixed in 2% PFA-2% glutaraldehyde in 50 mM cacodylate buffer at pH 7.4 for 2 h. Cells were fixed in 1% osmium tetroxide in 125 mM cacodylate for 30 min. Samples were dehydrated in solutions with gradually increasing the concentration of ethanol (50, 70, 95, and 100% three times) for 15 min each. Cells were included in epoxy resin (48.2% epon 812, 34% anhydride nadic methyl, 16.4% anhydride [2-dodecenyl] succinic, and 1.5% 2,4,6-tris [dimethylaminoethyl] phenol) for 48 h at 60 °C. After resin polymerization, in order to cut them, a heat shock was first applied to remove glass coverslips. To obtain sagittal sections of cells, the cutting surface was reoriented by preparing small blocks using a circular saw (Bronwill Scientific, USA) and sticking them on new ones. Ultra-thin cross sections (100 nm) were obtained using an automatic ultramicrotome (Ultracut-E Ultramicrotome, Reichert Jung, USA). The sections were stained by 5% uranyl acetate for 20 min and 4% lead citrate. The specimens were observed using a transmission electron microscope EM208 (FEI Compagny, Philips, Netherlands) operating at an accelerating voltage of 70 kV. Images were captured on argentic SO163 Kodak films.

### Confocal laser scanning microscopy

Confocal laser scanning microscopy (CLSM) observations were carried out on a Zeiss LSM 510 microscope using a 63× (Zeiss Achroplan) objective and with 0.4 μm z-section intervals. FITC fluorescence was detected after excitation at λ = 488 nm using a cut-off dichroic mirror of 488 nm and an emission band-pass filter of 505–530 nm (green emission). Alizarin complexone fluorescence was detected after excitation at λ = 543 nm using a dichroic mirror of 543 nm, and an emission long pass filter of 585 nm (red emission).

### Nanoparticle transfection

#### HCV replicating cell transfection

Huh7.5.1 cells chronically replicating HCV were propagated in complete medium (described above). CPNps were diluted in fresh medium to obtain 650, 520, 390, 260 and 130 ng of siRNA (final quantity) and added to the HCV replicating cells. Cells were lysed 3 days later and viral infection was assessed by measuring the luciferase activity. For each experiment, control transfections were performed using a mix of 12 nmol of πPEI polymer and 650 ng of siRNA (πPEI) and by transfecting cells with the different siRNAs using a commercially available transfection reagent (DF = Dharmafect, purchased from Dharmacon Inc.).

#### Kinetic

HCV replicating Huh7.5.1 cells were propagated in complete medium supplemented with 1% of DMSO to decrease cell proliferation. CPNps were diluted in medium plus DMSO to obtain 650, 390 or 130 ng of siRNA (final quantity) and added to the HCV replicating cells. Cells were lysed at 3, 6 or 10 days after CPNp transfection to measure HCV replication by luciferase assay.

For each experiment, a negative control transfection was performed using 12 nmol of πPEI polymer added in the culture medium of Huh7.5.1 cells and a positive control transfection was performed by transfecting siRNAs with a commercially available transfection reagent (Lipofectamine, purchased from Life Technologies™ (Carlsbad, CA, USA)).

### Cell viability assay

CPNp toxicity was assessed using PrestoBlue^®^ cell viability reagent purchased from Life Technologies™ (Carlsbad, CA, USA), according to the manufacturer’s instructions. Briefly, the PrestoBlue^®^ reagent was added to the cell culture medium of transfected or control Huh7.5.1 cells. After 1 h at 37 °C, absorbance was measured at 570 nm and 600 nm (reference wavelengths) using a microplate reader (Mithras LB 940, Berthold Technologies).

### Statistics

Data sets were analyzed using the Mann–Whitney test. *P* < 0.05 and 0.01 were considered statistically significant. Significant *p* values are indicated by asterisks in the individual figures: * = *p* < 0.05; ** = < 0.01.

## Conclusions

Our composite nanoparticles prepared by alternate deposition of siRNA and πPEI proved to be efficient in controlling HCV replication with up to 90% reduction over 10 days. Efficacy is directly related to the type of siRNA, the intracellular pathway targeted, and more interestingly the design of the deposition. Here, we show that the use of calcium phosphate particles improves the transfection efficacy of πPEI and confirm the works previously done by others using PEI. Our data pave the way for a better understanding of PEI transfection that is still believed to be the best candidate for synthetic transfection in the frame of therapeutic approaches. Moreover, the results presented here show that sequential siRNA deposition leads to a sequential release. Thus, these types of particles could be a useful tool for screening various pathways in the viral cycle in a time dependant assay to decipher robust targets for viral infection as well as cell circuits leading to cancer.

## Supplementary Material

† Electronic supplementary information (ESI) available. See DOI: 10.1039/c6tb01718e

ESI
