# Supplementary material for: Composite vector formulation for multiple siRNA delivery as a host targeting antiviral in a cell culture model of hepatitis C virus (HCV) infection†
Source: J Mater Chem B. Author manuscript; Available in PMC 2022 Aug 26. (PMC7613424; doi:10.1039/c6tb01718e)

This journal is © The Royal Society of Chemistry 2016

Supplementary informations

Composite vector formulation for multiple siRNA delivery as host targeting antiviral in a cell culture model of hepatitis C virus (HCV) infection

Results

Figure S1: Morphology and size of the (CPNp(πPEI/siRNA)$_{2.5}$) observed by TEM.

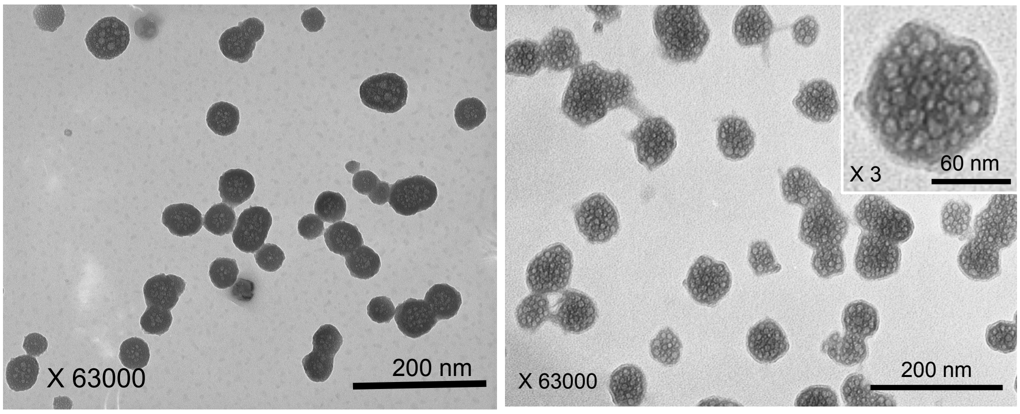

Figure S2: Measure of (CPNp(πPEI/siRNA)$_{2.5}$) in DLS at 1, 3 and 8 days at 4°C after coating.
Evolution of the size of (CPNp(πPEI/siRNA)$_{2.5}$) over time. Particle size after coating was recorded over a period of 8 days by DLS (day 1, 3 and 8). The diameter of the particles remains stable over time for at least 8 days in acetate buffer 5 mM pH = 5.5.

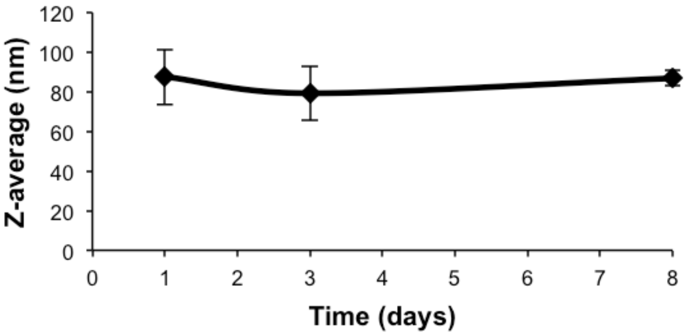

Figure S3: Electrophoresis of (CPNp(πPEI/siRNA)$_{2.5}$) or siRNA alone in water and serum.

The stability of surface coatings was assessed by a gel retardation assay. Nanoparticles were incubated in water or in fetal bovine serum for 1 hour and subjected to electrophoresis in a 2% agarose gel. No detectable siRNA release was observed from the (CPNp(πPEI/siRNA)$_{2.5}$) when the particles were incubated in water or complete serum. siRNA was deposited and migrated as a control.

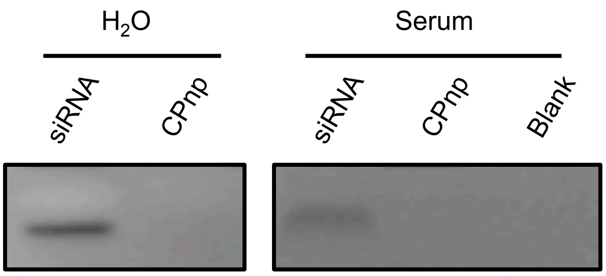

Figure S4: πPEI toxicity on Huh7.5.1 cells. Cell viability was measured by MTT test.

Huh7.5.1 cells viability after πPEI exposure was evaluated by the MTT (3-[4,5-methylthiazol-2-yl]- 2,5-diphenyl-tetrazolium bromide) assay. Briefly, Huh7.5.1 cells were grown in a 96-well plate (1x10^4 cells per well) and treated with several concentrations of πPEI. Twenty-four hour post-treatment, medium was discarded and cells were thoroughly washed with 200 μL of PBS. Fresh cell culture medium (200μL) + MTT (0.5 mg/ml) were added to each well and cells were incubated for 3h30 at 37 C and 5% CO_2. Medium was then carefully discarded and 100 μl of DMSO were added to each well forted 15 min at room temperature under orbital shaking. Color developed after the reaction was measured at 550 nm using Xenius microplate reader (SAFAS, Monaco).

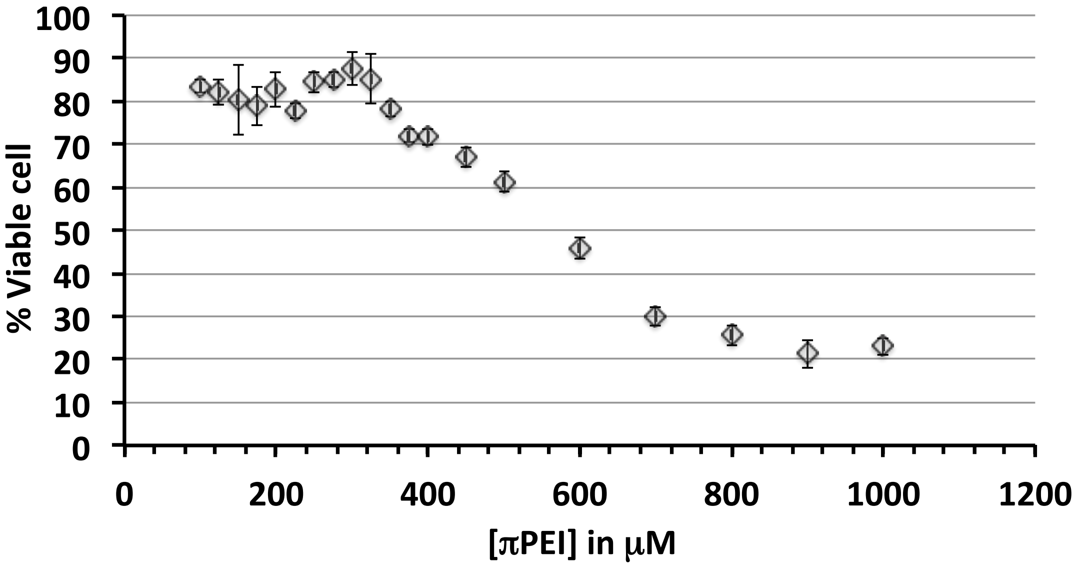

Figure S5: Effect of composite nanoparticles on HCV replication.

Chronically replicating HCV Huh7.5.1 cells were propagated in complete medium and transfected with CPNp coated with siHCV **(A)**, siRACK1 **(B)**, siCD81 **(C)**, siApoE **(D)** or a mix of siCD81/siRACK1 or siApoE/siRACK1 **(E)**. For each condition, particles were diluted in fresh medium to obtain 650 ng, 520 ng, 390 ng, 260 ng and 130 ng of siRNA (final quantity) and added to the HCV replicating cells. Cells were lysed 3 days later and viral replication was assessed by measuring luciferase activity (RLU). For each experiment, transfection controls were performed using the πPEI and DF (Dharmafect). Results are presented as % luciferase activity relative to non-transfected HCV replicating cells (Mock = 100%). Means ± SD from three **(C and D)** or two **(A, B, E and F)** independent experiments performed in triplicate are shown.

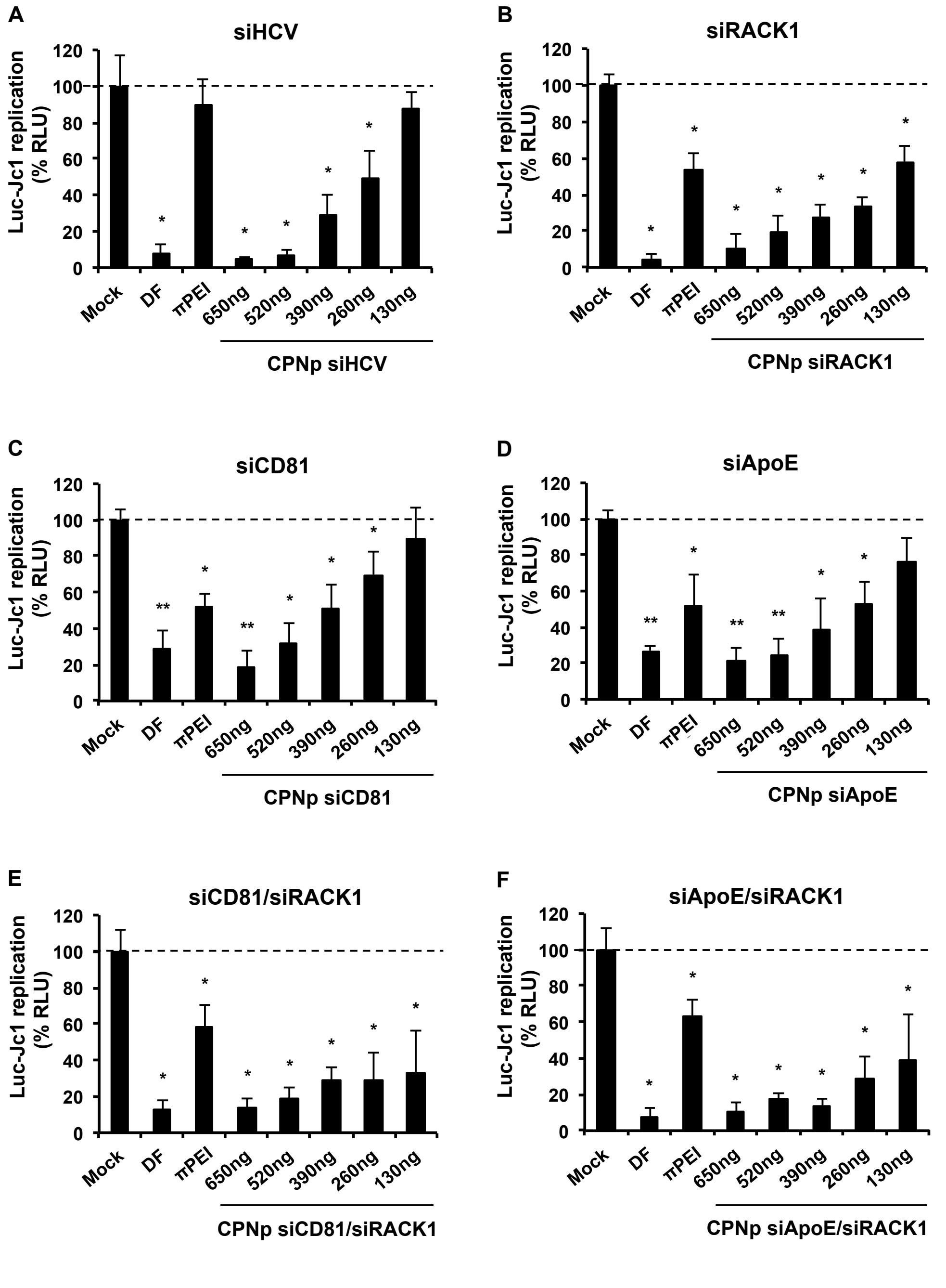

Supplement: ESI [file EMS151323-supplement-ESI.pdf]
